# Camp Treatment of Consumptives

**Published:** 1901-11

**Authors:** 


### CAMP TREATMENT OF CONSUMPTIVES.

The article from the pen of Dr. C. H. Wilkinson appearing in the present issue of the JOURNAL, is worthy of more than passing consideration. The writer points out the unsatisfactory claims of present methods for the cure of consumptives, as well as the abuses connected with the hotel resort management of such cases as usually practiced. He claims, what the JOURNAL has always contended, that in Western Texas atmosphere and elevation we have greater hopes for realizing satisfactory results with our patients than from any other mode of treatment known.

Dr. Wilkinson advocates the camp life treatment in this district for such invalids, and refers to the well known object lesson presented in the case of soldiers, who rarely ever contract consumption while in camp.

West Texas offers a magnificent field for such a venture, and the doctor believes that there is not only relief to be expected by the invalids who adopt his plan, but that in well selected and maintained camps for consumptives there is much money for the doctor who will undertake their management.

